# Editorial: Estrogen effects on fertility and neurodegeneration – classical versus non-classical actions

**DOI:** 10.3389/fendo.2023.1192671

**Published:** 2023-04-17

**Authors:** Zsuzsanna Nagy, Allan Herbison, Andrea Kwakowsky, Gergely Kovacs, Klaudia Barabas

**Affiliations:** ^1^ Institute of Physiology, Medical School, University of Pécs, Pécs, Hungary; ^2^ Department of Physiology, Development and Neuroscience, University of Cambridge, Cambridge, United Kingdom; ^3^ Pharmacology and Therapeutics, School of Medicine, Galway Neuroscience Centre, University of Galway, Galway, Ireland

**Keywords:** estrogen, estrogen receptor, GnRH, neurodegeneration, classical and non-classical actions

The gonadal steroid 17β-estradiol (E2) is the most potent form of estrogen with a broad spectrum of biological actions from fertility to neuroprotection. Studies during the last decades have provided a vast amount of data that have extended our understanding of the physiological importance of E2 in regulating a variety of tissues and organs, but many pieces of this intriguing puzzle remained to be elucidated (1). According to the classic paradigm, the cellular effects of E2 occur slowly: upon ligand binding cytoplasmic estrogen receptors (ERs) are translocated to the nucleus and regulate expression of target genes by binding to DNA sequences within hours or days (2). However, E2 can also exert rapid, non-classical effects in different types of cells including neurons. In response to their ligands, plasma membrane-bound estrogen receptors (ERs) are activated and E2 can change various cellular functions or modulate the transcription of several genes directly or indirectly by rapidly altering the activity of multiple signal transduction cascades (3).

This special issue is a collection of reviews and original research papers focusing on various aspects of the engagement of the hypothalamus-pituitary-gonadal axis in female reproductive functions and neurodegenerative diseases. The main female gonadal hormone E2 is a controversial molecule with the potential to have both beneficial and detrimental impacts on specific tissues. Non-classical actions of E2 in the brain are associated with advantageous effects like neuroprotection and enhanced cognitive functions, hence it is critical to explore further these mechanisms.

The article by Johnson et al. reviews the rapid, membrane-initiated estrogen signaling in female reproduction with a special focus on the interaction between the membrane-bound estrogen receptors (mERs) and the metabotropic glutamate receptors (mGluRs).

The study by Koppan et al. highlights the role of PACAP in the regulation of female reproductive functions including the GnRH-kisspeptin neuronal network, gonadal hormone production, follicular development, fertilization, embryonic/placental development, and maternal behavior. Results published by Barabás et al shed light on new angles of the GnRH-kisspeptin neural network in the modulatory effect of PACAP on the integrity of estrus cycle.

The article by Göcz et al. compares the estrogen-driven transcriptional responses of the two functionally different kisspeptin neuron populations that mediate the positive and negative feedback effects of estrogen. The RNA sequencing analysis uncovered new neuropeptides and mechanisms involved in the regulation of estrogen feedback.

Another study by Barabás et al based on a survey in Hungary observed the impact of COVID-19 pandemic and vaccination on the menstrual cycle but found no proof that the SARS-CoV-2 infection or vaccination were associated with menstrual cycle changes. The results, however, implicate that the increased levels of depression may cause the reported menstrual cycle abnormalities.

The paper by Rijal et al. investigates another aspect of the regulation of fertility. Their results show that the reactive oxygen species (ROS) acting as a signaling molecule affect fertility by directly modulating the excitability of gonadotropin-releasing hormone (GnRH) neurons.

Three articles in this Research Topic focus on the role of estrogen in neurodegeneration. As estrogen has been shown to have beneficial effects in the treatment of neurodegenerative diseases, two papers aimed to explore the mechanisms underlying the neuroprotective effects of estrogen. Farkas et al. demonstrated that female hormone depletion exacerbated the progression of Alzheimer’s disease-associated changes in the brain of a triple transgenic mouse model of Alzheimer’s disorder without causing cognitive behavioral symptoms. Kövesdi et al. analyzed the effect of estrogen on the activity of striatal cholinergic neurons that play a pivotal role in neurological disorders such as Parkinson’s and Huntington’s diseases. However, no evidence was found that estrogen alters the intrinsic properties of the striatal cholinergic neurons. The third article by Koszegi and Cheong is a mini review summarizing data collected on the potential use of estrogen analogues activating the non-classical pathways in the treatment of neurodegenerative diseases.

Finally, Makkai et al. contributes to the field of estradiol research by providing a deeper understanding of non-classical estradiol actions through investigation of receptor dynamics. By comparing two methods of calculating diffusion coefficients, the study suggests that Maximum likelihood-based estimation is a more reliable method for determining receptor movement, especially for cases with large localization errors or slow movements. This finding may have important implications for future research on membrane receptors and their function.

Overall, these articles provide new insights into the diverse effects of E2, illustrating the complexity of its actions and highlighting the many areas still to be explored in the field of neuroendocrine research.

## Author contributions

All authors listed have made a substantial, direct, and intellectual contribution to the work and approved it for publication.

